# Photoacoustic Flow Cytometry for Single Sickle Cell Detection *In Vitro* and *In Vivo*

**DOI:** 10.1155/2016/2642361

**Published:** 2016-09-01

**Authors:** Chengzhong Cai, Dmitry A. Nedosekin, Yulian A. Menyaev, Mustafa Sarimollaoglu, Mikhail A. Proskurnin, Vladimir P. Zharov

**Affiliations:** ^1^Arkansas Nanomedicine Center, University of Arkansas for Medical Sciences, Little Rock, AR 72205, USA; ^2^Division of Biochemical Toxicology, National Center for Toxicological Research, U.S. Food and Drug Administration, Jefferson, AR 72079, USA; ^3^Chemistry Department, M.V. Lomonosov Moscow State University, Moscow 119991, Russia

## Abstract

Control of sickle cell disease (SCD) stage and treatment efficiency are still time-consuming which makes well-timed prevention of SCD crisis difficult. We show here that* in vivo* photoacoustic (PA) flow cytometry (PAFC) has a potential for real-time monitoring of circulating sickled cells in mouse model.* In vivo* data were verified by* in vitro* PAFC and photothermal (PT) and PA spectral imaging of sickle red blood cells (sRBCs) expressing SCD-associated hemoglobin (HbS) compared to normal red blood cells (nRBCs). We discovered that PT and PA signal amplitudes from sRBCs in linear mode were 2–4-fold lower than those from nRBCs. PT and PA imaging revealed more profound spatial hemoglobin heterogeneity in sRBCs than in nRBCs, which can be associated with the presence of HbS clusters with high local absorption. This hypothesis was confirmed in nonlinear mode through nanobubble formation around overheated HbS clusters accompanied by spatially selective signal amplification. More profound differences in absorption of sRBCs than in nRBCs led to notable increase in PA signal fluctuation (fluctuation PAFC mode) as an indicator of SCD. The obtained data suggest that noninvasive label-free fluctuation PAFC has a potential for real-time enumeration of sRBCs both* in vitro* and* in vivo*.

## 1. Introduction

Sickle cell disease (SCD) is a genetic blood disorder caused by a single gene mutation in DNA that leads to production of abnormal hemoglobin (HbS) rather than normal hemoglobin (HbA) [1–6]. This process is accompanied by transformation of normal red blood cells (nRBCs) from a classic biconcave shape with a flattened center to an abnormally deformed sickle shape referred to here as sickle or sickled RBCs (sRBCs). These elongated rigid cells may occlude the microvasculature leading to infarctions. Deoxygenated HbS may precipitate and polymerize inside RBCs, thus enhancing their sickling [7]. Manifestations of SCD range from asymptomatic to life-threatening conditions [2, 3]. Stem-cell transplantation and gene therapy are promising the hope of a SCD cure [3, 4]. SCD diagnostics are well established including high-performance liquid chromatography, electrophoresis, mass spectrometry, and clinical quantification tests [8–11]. Nevertheless, most of these methods are time-consuming and cannot be applied for real-time monitoring of treatment efficiency and for out-of-bed predictions of vasoocclusive episodes (crisis) accompanied by acute debilitating pain or in some cases stroke, especially in children [10, 11]. Most episodes of SCD crises are unpredictable. Thus, searches for diagnostic methods to control SCD stages and therapy efficiency are still ongoing.

We propose that one of the possible solutions to identify and enumerate sRBCs* in vitro* and* in vivo *is to use the principle of photothermal (PT) and photoacoustic (PA) flow cytometry (PTFC/PAFC) and microscopy (PTM/PAM). These techniques are based on detection of the absorbed energy transformed through nonradiation relaxation into heat and accompanied acoustic waves, respectively [12–16]. In particular, the tremendous potential and safety of* in vivo* PAFC have already been demonstrated in many applications including detection and enumeration of circulating nRBCs, tumor cells (CTCs), bacteria, nanoparticles, and recently malaria parasites with the sensitivity, which is 1000 times better compared to the existing methods [17–23]. A clinical* in vivo* PAFC-based device has demonstrated label-free detection of CTCs directly in the bloodstream of melanoma patients with the detection limit of one CTC in 300 mL of blood that is ~100-fold better than that in CTC assays* ex vivo* [22]. PTM and PAM also demonstrated a great potential for high resolution and super resolution imaging of nonfluorescent cells, dyes, and nanoparticles [17–21]. However, little progress was made in exploring the potential of PT and PA techniques for basic and clinical SCD-related research. We demonstrate here that these techniques may be used for detection and identification of sRBCs using the dependence of PT and PA signal parameters on the optical and morphological distinctive features of sRBCs.

## 2. Materials and Methods

### 2.1. The Principle of PT and PA Techniques

**Figure 1:**
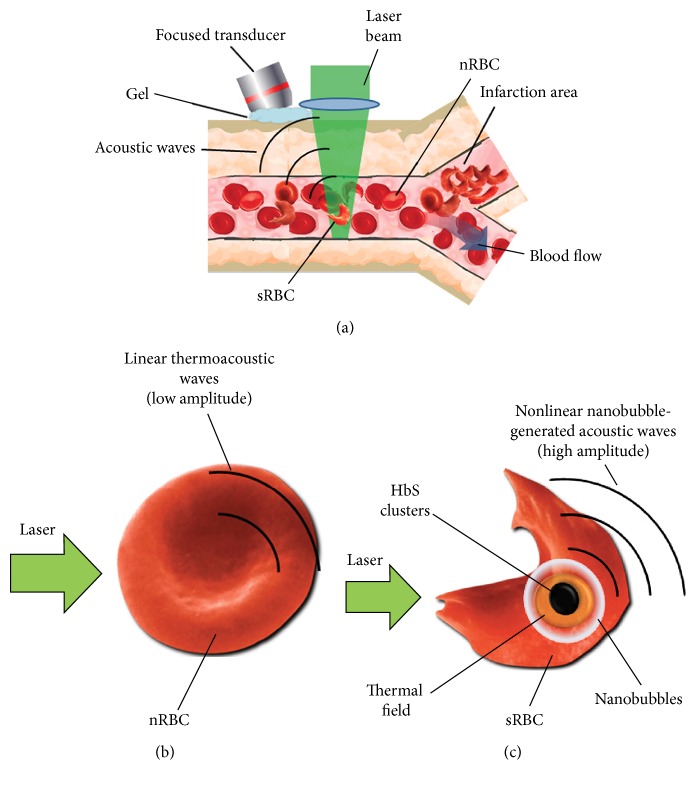
Principles of PA monitoring of sRBCs during SCD crisis. (a) Schematic of PAFC. (b, c) Principles of linear (b) and nonlinear nanobubble-based (c) PA detection of relatively homogenous HbA and clustered HbS distribution in normal and sickled cells (nRBCs and sRBCs), respectively.

The differentiation of sRBCs with HbS clusters from nRBCs with HbA using* in vivo* PAFC was based on irradiation of selected vessels by laser pulses (Figure 1) followed by detection of linear (Figure 1(b)) and especially nonlinear (Figure 1(c)) acoustic waves with an ultrasound transducer placed on the skin near laser beam [22]. Sickled cells may form HbS clusters dense enough to cause local overheating and formation of nanosized air bubbles. The nonlinear enhancement of PA signals from such cells, appearing as transient high amplitude PA signals, may allow detection of cell sickling even in the presence of other sRBCs having homogenous intracellular HbS distribution. PT microscopy method was implemented for high resolution imaging of intracellular Hb distribution and used two lasers (pump-probe) for probing single cells. Probe beam was sensing pump beam-induced changes in refractive index of a medium (thermal lens mode) [12, 17, 18].

### 2.2. Integrated PTM and PAM Platform

**Figure 2:**
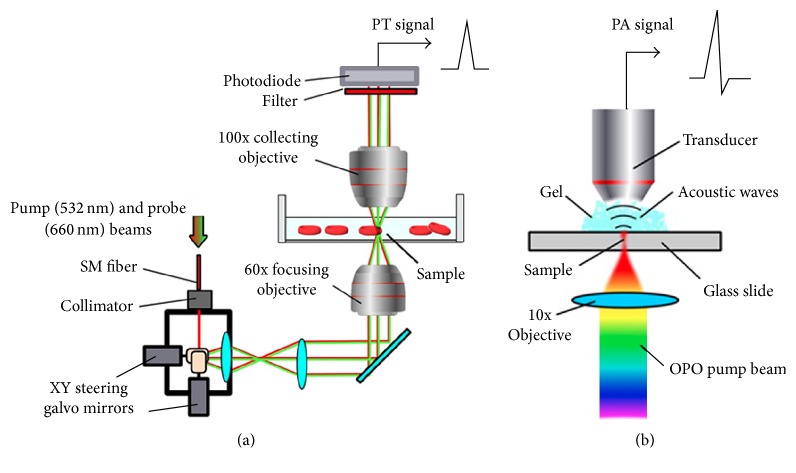
Schematics of PT and PA instrumentation. (a) Laser scanning confocal two-beam (pump-probe) PT microscopy (PTM). (b) PA spectrometer with an automatic spectrally tunable laser.

The integration of spectral PTM and PAM was performed on an Olympus IX81 inverted microscope platform (Olympus America, Center Valley, PA). PT and PA spectra were obtained with a tunable optical parametric oscillator (OPO, Opolette HR 355 LD, OPOTEK, Inc., Carlsbad, CA) [17]. The imaging system was based on steering laser beam by a pair of XY steering galvo mirrors (6215H, Cambridge Technologies, Lexington, MA) from a pulsed laser (wavelength, 532 nm; pulse width, 5 ns; pulse repetition rate, 1–10 kHz; LUCE, Bright Solutions, Italy). A continuous wave (CW) laser diode with wavelength of 660 nm (Power Technology, Alexander, AR) was used in PT mode as a probe beam to control changes in the refractive index caused by the pump beam (Figure 2(a)). Both laser beams were focused into the sample using a 60x objective (DPlan 60, Olympus, Inc.). The probe beam was collected after the sample by the second immersed 100x objective (LUMPlanFl 100, NA 1.00, Olympus, Inc.). Defocusing of the probe beam was detected using a fast photodiode (PDA10A, Thorlabs Inc.). The probe beam intensity changes were measured to obtain transmission images. The 3D reconstruction of absorption profile was made using ImageJ software using a stack of PT images acquired along *z*-axis by displacing focusing objective in 0.1 *μ*m steps. In PA spectroscopy and imaging modes laser beams were focused by 10x objective (Plan Achromat, 0.25 NA, Olympus, Inc.) and acoustic waves were detected by focused ultrasound transducer (V316-SM, 20 MHz; focal length: 12 mm, Olympus NDT, Waltham, MA) placed on microscopic slide* in vitro* or mouse ear* in vivo* (Figure 2(b)). PA signals were preamplified (amplifier model, AG-2010, ONDA Corp., Sunnyvale, CA) and recorded. PA signals were normalized to OPO pulse energy to calculate the PA image and spectrum of the sample. A high-speed analog-to-digital converter board (PCI-5124, National Instruments Corp.) with customized software (LabVIEW, National Instruments) was used to acquire signals in both PA and PT modes.

### 2.3. *In Vitro *and* In Vivo *PA Flow Cytometry (PAFC)

PAFC was described in detail elsewhere [22]. PAFC system was equipped with high pulse repetition rate lasers with wavelength of 532 nm and 820 nm (LUCE 532 and LUCE 820, Bright Solutions). Acoustic waves were detected using a 3.5 MHz ultrasound transducer (Imasonic Inc., Besancon, France). The electrical signals were then amplified and recorded as described above.

*In vitro* PA detection of single RBC in flow was performed using a 532 nm laser in sodium citrate stabilized blood samples diluted by PBS to 1 : 10^4^ to ensure presence of single cells in the detection volume to avoid temporal signal overlapping. Samples were pumped through 50 *μ*m (i.d.) quartz capillaries at flow velocity of 1 mm/s followed by acoustic wave detection through the capillary walls.* In vivo* PAFC was performed directly in 40–60 *μ*m ear veins of anesthetized mice using 820 nm laser.

### 2.4. Animal Model

*In vivo* experiments involved nude mice (nu/nu), black C57BL/6 mice, and genetically modified mice expressing human sickle hemoglobin (STOCK Hbatm1Paz Hbbtm1Tow Tg[HBA-HBBs]41Paz/J mice [Berkeley mice]), further referred to as “SCD mice.” All the studies were performed in accordance with protocols approved by the Institutional Animal Care and Use Committee, University of Arkansas for Medical Sciences. Animals were anesthetized with isoflurane (1.5% in pure oxygen), and placed on a temperature-controlled stage (37°C). For each animal we selected appropriate vessels (50–70 *μ*m-in-diameter ear veins). The laser beam was positioned over the vessel and PA signal traces were recorded for at least 10 min. Nonlinear PA signals were acquired from the vessels by increasing the laser energy in steps. Signal traces were recorded for 1 min at each level of laser energy. In order to prove the feasibility of PA detection of HbS, ~100 *μ*L whole blood from SCD mice was intravenously injected into the nude mouse and changes in PA signal traces were recorded for up to 20 min. To enhance the presence of sRBCs through HbS polymerization in the deoxygenated RBCs in SCD mice, we triggered hypoxia in selected mice through gradual increase of isoflurane concentration from 1.5% up to 4-5% level. High anesthetic concentration decreases blood pressure [24]. After 2-3 min at 5% isoflurane concentration, blood flow velocity usually decreased to a full stop. Upon observing the nonlinear PA signals in mice, the isoflurane concentration was returned back to normal levels to restore blood flow.

### 2.5. Preparation of Blood Samples* In Vitro*

Whole blood samples of donor SCD mice were collected from tail vein and stabilized with 3.2% sodium citrate. Blood smears fixed with methanol were studied using 35 mm-in-diameter glass-bottom dish. For UV-Vis spectral analysis, the Hb solution with physiologically relevant total Hb concentration (110–160 g/L) was prepared by dissolving Hb powder (H0392 and H0267, for sickle and normal Hb, respectively, Sigma-Aldrich, Co., St. Louis, MO). For spectral analysis, all blood samples were oxygenated for 10 min by gentle inversion of the sample tubes. The blood oxygenation was verified using optical absorption spectra and existing reference spectra [25]. To prove the significance or insignificance of spectral changes, the difference in a tested spectrum for each sample were compared with the sum of errors under these conditions.

Human blood samples stabilized with 3.2% sodium citrate were obtained from healthy volunteers at the Fundamental Medicine Department of the Moscow State University (MSU), Russia. All the experiments were approved by MSU IRB committee.

## 3. Results

### 3.1. PA Spectroscopy of Hemoglobin Subtypes

**Figure 3:**
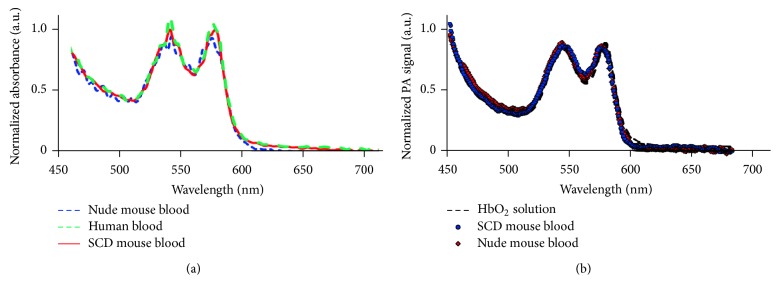
Absorption and PA spectra of HbS and HbA alone and in RBCs. (a) Conventional absorption spectroscopy of nude mouse blood, SCD mouse blood, and human blood. Sample absorption was normalized to absorption at 450 nm. (b) Single cell PA spectroscopy using spectrally tunable automatic laser optical parametric oscillator (OPO). The spectrum of HbO_2_ was acquired using conventional spectroscopy from 90% oxygenated hemoglobin solution.

We performed conventional optical photometric/absorption measurements for Hb solution and blood from both healthy control and genetically modified SCD mice expressing human HbS. The comparison with a reference spectrum of the oxygenated Hb (HbO_2_) shows that, in all the samples, the content of HbO_2_ was up 90%. The experimental data (Figure 3(a)) demonstrated a very good agreement between the spectra of nude mouse blood, HbS containing blood, and human blood with less than 2% average deviation from the human blood spectrum selected as reference. The differences in total light absorbance of the samples were associated with different hematocrit levels of healthy and SCD blood [26]; thus, absorption spectra were normalized for further comparison. Additionally, we acquired PA spectra in linear PAM mode at relatively low energy fluence (20 mJ/cm^2^) from a small volume (down to a single cell level) of sickled and normal cells to avoid averaging of HbA and HbS in a larger sample and to minimize the influence of light scattering into cells. PA spectra of both normal and SCD hemoglobin matched well with the measured absorption spectrum of HbO_2_ solution and with oxyhemoglobin spectrum (Figure 3(b)) in accordance with the previously published HbA and HbS spectra [25]. Thus, no significant differences in conventional absorption spectra of HbA, HbS, and PA single cell spectra were observed except slight differences in the spectral range of 590–660 nm in some measurements. However, this feature is not enough at current stage for spectral identification of sRBCs and requires additional verifications in the future.

### 3.2. High Resolution PT Imaging of Normal and Sickled Cells

**Figure 4:**
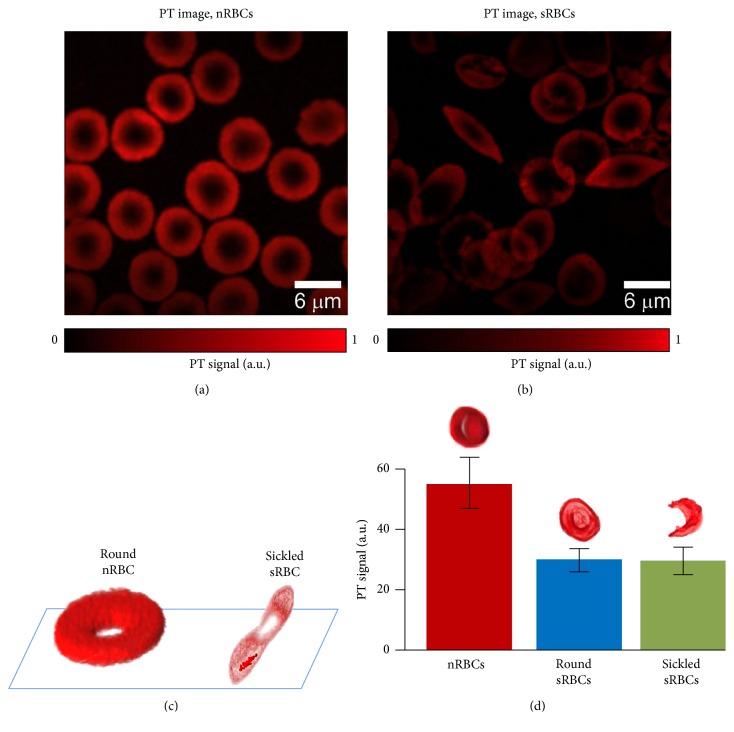
High resolution PT imaging of nRBCs and sRBCs* in vitro*. (a, b) PT images of nRBCs and sRBCs. (c) 3D reconstruction of absorption profile based on z-stack of multiple PT images for a healthy nRBC (left) and a single sickled sRBC (right). (d) PT signal amplitudes from individual nRBCs and sRBCs with round and deformed shape. Laser parameters: wavelength, 532 nm; energy fluence, 20 mJ/cm^2^; laser beam diameter, 0.5 *μ*m.

Confocal PTM imaging [17] is uniquely suited for mapping intracellular Hb distribution of live RBCs. As we demonstrated previously, conventional transmission microscopy may be hindered by light scattering artifacts and low absorption sensitivity [12, 17] because of short optical pathway in single cells. PT images were free of light scattering effects (Figures 4(a) and 4(b)). High resolution confocal PTM demonstrated the ability for high resolution (0.3–0.5 *μ*m) imaging of live sRBCs on a glass surface* in vitro* without the use of any trapping or cell immobilization techniques. PT data revealed the existence of elongated HbS clusters oriented mostly along the main axis of the cell (Figure 4(b)). 3D reconstruction of PT signals for nRBCs and sRBCs (Figure 4(c)) confirmed that HbS is tightly condensed in sRBCs with the appearance of HbS clusters with higher local absorption compared to intracellular background. Because PT signal amplitudes are proportional to cellular absorption, the presented 3D images (Figure 4(c)) reflect the cellular absorption distribution. Thus, PT mapping of intracellular absorption distribution revealed more profound heterogeneous absorption profile in sRBCs especially in sickle cell shape. However, PT signals averaged within whole cells in linear mode at low laser energy fluence demonstrated significantly (2–4-fold) lower amplitudes from sRBCs than from nRBC (Figure 4(d)) suggesting lower Hb content in sRBCs. The difference in PT signal amplitudes from round and sickled sRBCs was not significant; however, in some cases, signal amplitudes were a little lower from sickled sRBCs.

### 3.3. Nonlinear PA Imaging of Normal and Sickled Cells* In Vitro*

**Figure 5:**
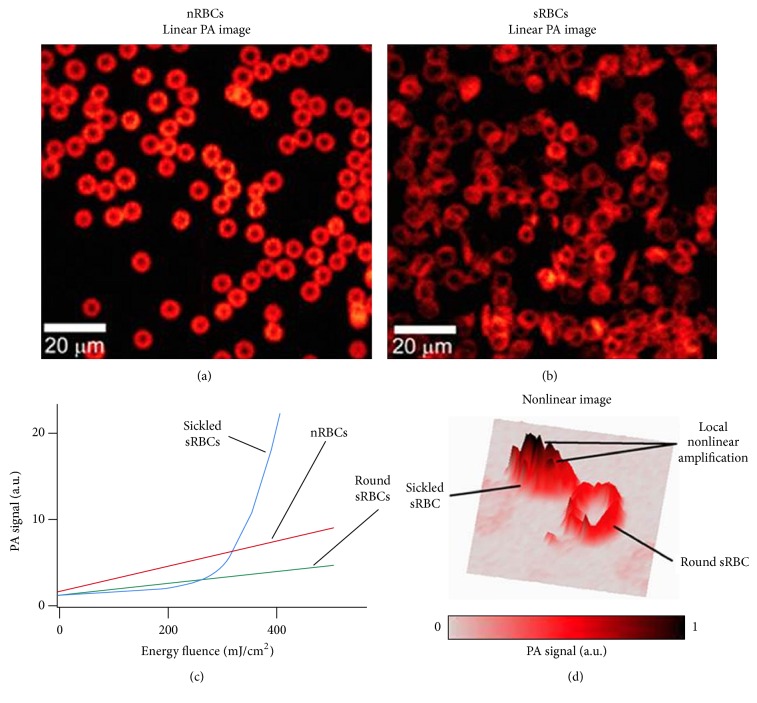
PA imaging of nRBCs and sRBCs* in vitro*. (a, b) PA images of nRBCs (a) and sRBCs (b) in glass slides. (c) The dependence of PA signal amplitudes on laser energy fluence for single RBCs with different states and shapes demonstrating linear behavior for nRBCs and round sRBCs and nonlinear signal amplification for sRBCs with deformed shape. Data represents average PA signals from 50 cells. (d) PA images of two sRBCs at relatively high energy fluence demonstrating more profound nonlinear nanobubble-based amplification of PA signals from sRBC with deformed shape. Laser parameters: wavelength, 532 nm (a–d); energy fluence, 50 mJ/cm^2^(a, b) and 400 mJ/cm^2^(d).

The discovery of HbS clusters with high local absorption in sRBCs led to our hypothesis that spatially selective laser overheating of HbS clusters can generate nanobubbles as PA signal amplifiers [27] around HbS clusters only. As a result, despite lower average absorption in sRBCs, nanobubbles can significantly increase PA signal amplitude above nRBCs background, thus making the selective PA detection of sRBCs in nonlinear mode possible. To verify this hypothesis, we obtained initially linear PA images of nRBCs and sRBCs (Figures 5(a) and 5(b)) which were very similar to the linear PT images (Figures 4(a) and 4(b)). They showed many cells with abnormal sickled shapes. To estimate the influence of laser energy in nonlinear mode, we measured PA signal amplitudes from individual cells as a function of laser energy fluence for nRBCs, round and sickled sRBCs (Figure 5(c)). All nRBCs and some sickled sRBCs with round shape provided linear PA signals up to energy fluence of 600–800 mJ/cm^2^ at wavelength of 532 nm and the PA signal amplitudes from sRBCs were lower than those from nRBCs which is in line with PT data (Figure 4(d)). However, some sRBCs with asymmetrical sickle-like shapes provided a dramatic nonlinear increase in PA signal amplitudes when energy fluence exceeded 200 mJ/cm^2^ (Figure 5(c)). On average, the sickle shaped sRBCs demonstrated 3–5-fold signal enhancement which could make them detectable among nearby round sRBCs and nRBCs (Figure 5(d)). It is important that the cells remained visually intact after detection. Nonlinear PA effects caused no damage to cell membrane during the experiment or 30 min afterwards.

### 3.4. *In Vitro* PAFC of Normal and Sickled Cells

**Figure 6:**
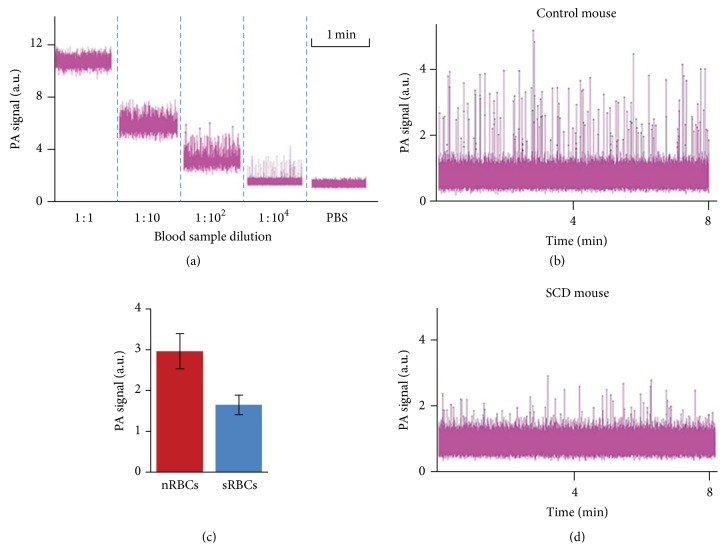
*In vitro *PAFC of sRBCs. (a) PA signal amplitudes from nRBCs of SCD mouse, flowing in a capillary tube at different dilutions showing completely overlapped (1 : 1) and nonoverlapped (1 : 10^4^) signal peaks from individual sRBCs. (b, d) Typical PA signal traces with nonoverlapping peaks from individual nRBCs of a nude mouse (b) and sRBCs of a SCD mouse (d). (c) Averaged PA signal amplitudes from nRBCs and sRBCs in (b, d).

To confirm the findings above with cells in flow, blood samples from normal and SCD mice were analyzed using* in vitro* PAFC at flow velocity of 1 mm/s in capillary tube of 50 *μ*m diameter. Because of the temporal overlapping of PA signals from individual RBCs at normal blood concentration, the blood samples were sequentially diluted which led to reducing the average PA signal background and increasing the background fluctuation due to a lower number of cells in the detection volume (Figure 6(a)). In highly diluted blood samples, we observed transient PA signals above noise corresponding to single cells passing the detection zone. Dilution of the sample over 10^4^ decreased both PA background and number of peaks but not peak amplitudes, confirming the detection of individual cells. Under these condition, PA signal amplitudes from single nRBC from healthy mice (Figures 6(b) and 6(c)) were 2-3 times higher than those from SCD mice (Figures 6(d) and 6(c)) which confirmed previous PT (Figure 4(d)) and PA (Figure 5(c)) image data. This finding indicates that there is a higher total Hb content per cell in healthy mice and the possibility for sRBCs identification at least* in vitro* based on the analysis of signal amplitude.

### 3.5. *In Vivo *PAFC Monitoring of Circulating Sickled Cells

**Figure 7:**
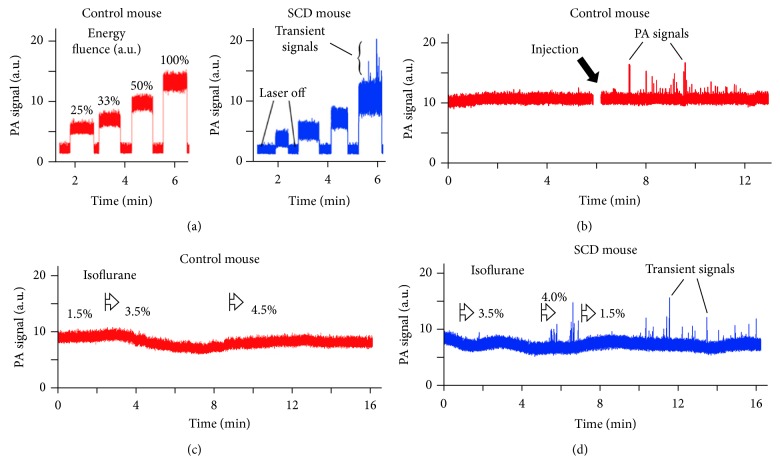
*In vivo* PAFC of sRBCs in SCD mice. (a) Typical* in vivo* PA signal traces from mouse ear microvessels (veins) at different laser energy fluence (relative units) for control nude (left) and SCD (right) mice. (b)* In vivo* PA signal trace after intravenous injection of 100 *μ*L of SCD mouse blood into healthy mouse blood circulatory system. (c, d)* In vivo* PA signal traces in normal nude mice (c) and SCD mice (d) during isoflurane induced hypoxia. Laser parameters: wavelength, 820 nm (a–d); laser energy fluence, 10–600 mJ/cm^2^(a) and 500 mJ/cm^2^ (b–d).

To compare data of PAFC* in vitro* and* in vivo*, we monitored PA signals from ear veins (50–70 *μ*m in diameter) in healthy and in SCD mice. At increasing laser energies and similar blood vessel parameters, we observed three phenomena: (1) lower PA background signals from the vessels in SCD mice, (2) stronger PA background signal fluctuation in SCD mice, and (3) appearance of transient PA signal peaks in SCD mice at relatively high laser energy (Figure 7(a)). These findings are consistent with our PT and PA data* in vitro* showing lower PA signal amplitudes from individual sRBCs, more heterogonous PA signal amplitude distribution, and nonlinear PA signal amplification from sickled RBCs, respectively (Figures 4(d), 5(c), 5(d), 6(b), 6(d), and 6(c)). High pulse repetition rate (10 kHz) laser operating at wavelength of 820 nm was used to estimate clinical perspectives of a near-infrared (NIR) laser with deeper skin penetration compared to laser at 532 nm. Moreover, in NIR range at 820 nm, blood oxygenation does not affect PA signals, as both Hb and HbO_2_ have similar absorption levels. Although PA background may increase due to skin pigments in SCD mice compared to nude mice, the PA signals from similar vessels were higher than those from skin which allowed observation of vessel-related phenomena without strong influence of skin pigmentation. We emphasize that, at relatively high energy fluence of 300–400 mJ/cm^2^, we observed both enhancement of PA background fluctuation and transient PA peaks in SCD mice (Figure 7(a), right) which were not observed in healthy mice (Figure 7(a), left). These signals were likely associated with previously discovered nonlinear amplification of the PA signals in HbS clusters. No tissue or vessel damage was observed even after 30–40 min long monitoring.

To verify these findings, under the same condition as in previous study, 100 *μ*L blood sample collected from SCD mouse was injected directly into blood circulation of a healthy mouse. We observed transient PA peaks above noise previously absent in PA signal trace (Figure 7(b)). These PA peaks lasted only for 3–10 minutes after injection suggesting their fast clearance in healthy mice. We also tested PA detection in control C57BL/6 mice also having pigmented skin and similar genetic make up to the SCD mice but expressing no HbS. PA traces for C57BL/6 mice (Figure 7(c)) were similar to those acquired from control nude mice (Figure 7(a)) and featured no transient PA signals.

To demonstrate feasibility of PA monitoring of early manifestations of SCD diseases, we decreased mouse blood oxygenation to enhance polymerization of HbS by using higher concentration of anesthesia drug (isoflurane) which led to additional amount of sickling sRBCs and HbS clustering. Isoflurane concentration increased from typical 1.5% to 4-5% level (Figures 7(c) and 7(d)) which was sufficient to dramatically decrease breathing rate and blood flow pressure. The use of 5% isoflurane for more than 5 min was avoided as it could slow breathing enough to cause death through hypoxia. The transient PA signals were observed only for SCD mice at ~4% anesthetic concentration, while no peaks were observed for healthy mice even at the 5% level (kept until complete termination of the blood flow through a vein). Nonlinear transient PA signals were detectable in SCD mice for up to 10 min after decreasing isoflurane concentration down to normal levels (1.1–1.5%) confirming that for some cells depolymerization of HbS is slow even after restoration of oxygen supply. For both healthy and SCD mice, an increase in anesthetic concentration over 3.5% resulted in decreased blood flow velocity and decreased vessel diameter. A decrease in vessel diameter together with blood deoxygenation resulted in decrease of PA signals from the vessel at high isoflurane concentration. In many cases fluctuations of PA signal traces were higher in SCD mice compared to normal mice, thus confirming the ability of “fluctuation” PAFC to distinguish SCD disease.

## 4. Discussion

In this work, we demonstrated for the first time that PT and PA techniques with spectroscopic, imaging, and cytometric schematics and modes can provide the identification of both round and sickled sRBCs among nRBCs in linear and especially nonlinear mode. We also showed the possibility for noninvasive PA monitoring of HbS polymerization in RBCs directly in blood flow. Mouse ear veins were selected in this work as an optimal animal model with a thin (100–200 *μ*m) skin layer. As expected, normal and sickle cells (nRBCs and sRBCs) have different size and shape (Figures 4 and 5). In particular, on average, nRBCs had a symmetrical biconcave shape with an average diameter of ~6.8 *μ*m. The sRBCs were less symmetric than nRBCs with multiple cells in transition from symmetrical biconcave to asymmetric sickle-like shape with an average diameter of 7.8 *μ*m. In many samples, we also observed the presence of background PA signals from the medium between sRBCs which was not observed in nRBCs samples probably due to release of HbS from sRBCs (e.g., due to specific membrane property or local membrane damage).

Many sickle shaped sRBCs with typical concentration in SCD mouse model of 10% provided significant signal enhancement of up to 5–10-fold (Figure 5(c)). Such PA signal amplification opens the opportunity for selective detection of sickled sRBCs among round sRBCs and even nRBCs* in vivo* through monitoring of enhanced PA signal background fluctuation and the appearance of transient PA signal peaks (Figures 7(a)–7(d)). For instance, nonlinear PAFC requires relatively high level of laser energy which may slightly exceed the laser safety standard levels. This may lead to the damage of RBCs containing Hb clusters. Still, the cavitation enhancing PA signal from sickled sRBCs usually is very local (within a few hundred nanometers) and would not necessarily damage cells. One of the potential solutions to reduce laser fluence would be to use picosecond or sub-nanosecond (200–600 ps) laser pulses for more effective generation of nonlinear PA signals from clustered HbS [27]. With the current nanosecond (10–30 ns) range, we did not observe any tissue or vessel damage using clinically promising high pulse repetition rate (10 kHz) laser with a wavelength of 820 nm. High pulse repetition rate additionally increased the sensitivity of PAFC by averaging of PA signals.

*In vivo* detection of sRBCs opens new perspective for diagnosis and monitoring of the SCD disease and especially prediction and potentially prevention of SCD crisis episodes during blocking of small vessels by rigid sRBCs (Figure 1(a)) by well-therapy. Specifically, PAFC technology may provide an early warning and/or indication for drug administration or blood transfusion and can control the efficiency of these procedures. In clinical settings, PA techniques may be performed on easily accessible capillaries in the human ear, hand, and nail bed [28]. Sickling of cells in the microvascular bed may have much higher predictive value compared to venous blood analyzed here. In this case, PA imaging system may provide a large field of view for simultaneous detection of cells in multiple capillary bed vessels. Further studies are needed to make sure that PAFC system is able to distinguish single sRBCs from sRBC cluster or nRBC aggregates.

Spectral identification of HbS from HbA using absorption signatures is still under debate [29, 30]. In current study, PA spectroscopy of single cells confirms close match between absorbance of nRBCs and absorbance of sRBCs. Most spectra acquired from blood samples and hemoglobin solutions were very close to each other leaving a little possibility to distinguish forms of hemoglobin using PA spectral data in visible spectral range, at least* in vivo*.

Nevertheless, polymerization of Hb inside sRBCs opens a perspective for identification of sRBCs through monitoring cell shape and/or analysis of Hb distribution inside individual cells. This may simplify testing at the bedside through simple cell shape analysis without sample preparation. We confirmed our previous findings [18, 22] that high spatial heterogeneity of PA signals from RBCs may serve as an indicator of Hb clustering. Compared to conventional optical microscopy, PT and PA imaging has a higher sensitivity allowing rapid screening to visualize the shape and size of RBCs in fast flow. Previously, we demonstrated high-speed PT imaging of cells using a single pulse PT microscopy [20] capable of visualizing Hb content of cells in flow* in vivo* and* in vitro* and at speeds up to 2 m/s.

The ability of PT and PA microscopy to map hemoglobin absorption distribution in individual RBCs may be used to study polymerization of Hb* in vivo* at a subcellular level. Using our novel PT-lifetime imaging beyond the diffraction limit (size-dependent PT signal shape reflecting cooling time and heat transfer in the sample), we previously demonstrated PT imaging and resolving of local Hb clusters ranging in size from 50 to 300 nm inside live RBCs [12]. Here, we show that PT data may be indeed used to build a model of Hb clusters inside sRBCs to study Hb polymerization. High resolution confocal PT microscopy demonstrated that, inside live sRBCs, HbS forms dense aggregates. Thus, this and similar techniques can be considered as new research tools to provide insights on SCD disease progression and correlation of individual cell properties (e.g., mechanical and chemical) with the assembly of Hb inside the cell. Moreover, multicolor PT and PA imaging using different laser wavelengths may provide additional data on Hb oxygenation and composition (e.g., meta-, carboxy-, and nitroso-). As already mentioned, we have discovered that polymerization of HbS may increase nonlinear PA signals in sRBCs and not in nRBCs. This is similar to previously discovered nonlinear nanobubble-related PA signal amplifications in melanin aggregates in melanoma cells [27] and recently in hemozoin clusters in malaria parasite-infected RBCs [23]. Our current finding indicates that nonlinear amplification of PA signals in sRBCs takes place at laser energy fluence when nRBCs generate only linear PA signals.

Further improvement of PAFC technology can be achieved by cell focusing directly in the bloodstream [31] to exclude PA signal overlapping at high sRBCs concentration or combination of PAFC with optical clearing procedure of skin to reduce several times laser energy losses in tissue due to light scattering phenomena [28]. Recently, we have tested a portable hand-worn PAFC prototype with small high pulse rate lasers which can be used for noninvasive, label-free control of SCD disease stage (see references in [28]).

We have to mention a few drawbacks of the selected animal models. First, our proof-of-concept experiments were performed using a homozygous model of SCD. In the homozygous model, the chances of finding Hb clusters with high nonlinear PA contrast are dramatically higher than in the hemizygous model, but such a model does not represent human traits well enough. Thus, focusing more on a clinically relevant hemizygous model will be essential after optimization of PA detection using the mice model selected here. Secondly, most of the control experiments were performed in nude mice that are different from the SCD model. A wider use of mice with a genetic makeup similar to SCD mice may provide additional information on sensitivity and presence of false positive results in PA detection. Finally, the biological data acquired in a model system may be influenced by total hemoglobin and hematocrit levels. In this case, both the conditions for sRBCs sickling and PA detection would change.

In conclusion, we believe that a simple low-cost microchip-based system may be designed for such screening using compact laser sources and cell-phone-like camera for detection of PT and PA signals from sRBCs. This may simplify SCD diagnosis in developing countries, where a clinical base for detection of HbS is not yet available, and high cost of genetic tests hinders their application.
